# Machine learning approach yields epigenetic biomarkers of food allergy: A novel 13-gene signature to diagnose clinical reactivity

**DOI:** 10.1371/journal.pone.0218253

**Published:** 2019-06-19

**Authors:** Ayush Alag

**Affiliations:** The Harker School, San Jose, CA, United States of America; Institut de genomique, FRANCE

## Abstract

**Background:**

Current laboratory tests are less than 50% accurate in distinguishing between people who have food allergies (FA) and those who are merely sensitized to foods, resulting in the use of expensive and potentially dangerous Oral Food Challenges. This study presents a purely-computational machine learning approach, conducted using DNA Methylation (DNAm) data, to accurately diagnose food allergies and potentially find epigenetic targets for the disease.

**Methods and results:**

An unbiased feature-selection pipeline was created that narrowed down 405,000+ potential CpG biomarkers to 18. Machine-learning models that utilized subsets of this 18-feature aggregate achieved perfect classification accuracy on completely hidden test cohorts (on an 8-fold hidden dataset). Ensemble classification was also shown to be effective for this High Dimension Low Sample Size (HDLSS) DNA methylation dataset. The efficacy of these machine learning classifiers and the 18 CpGs was further validated by their high accuracy on a large number of hidden data permutations, where the samples in the training, cross-validation, and hidden sets were repeatedly randomly allocated. The 18-CpG signature mapped to 13 genes, on which biological insights were collected. Notably, many of the FA-discriminating genes found in this study were strongly associated with the immune system, and seven of the 13 genes were previously associated with FA.

**Conclusions:**

Previous studies have also created highly-accurate classifiers for this dataset, using both data-driven and *a priori* biological insights to construct a 96-CpG signature. This research builds on previous work because it uses a completely computational approach to obtain a perfect classification accuracy while using only 18 highly discriminating CpGs (0.005% of the total available features). In machine learning, simpler models, as used in this study, are generally preferred over more complex ones (other things being equal). Lastly, the completely data-driven methodology presented in this research eliminates the need for *a priori* biological information and allows for generalizability to other DNAm classification problems.

## Introduction

Food Allergy (FA) is a specific immune response that occurs upon exposure to a particular food [1]. FA affects around 8% of children and 3-5% of adults [2][3] and poses high risks: around 40% of affected children experience anaphylaxis, a severe and potentially life-threatening reaction [4][5]. Equally worrisome is the fact that both the number of FA-affected people and the number of food-related anaphylactic events are increasing [6]. The financial costs of FA are significant as well, with an estimated price of $4,184 per year per affected child [7].

Current laboratory procedures used to detect food allergies, such as allergen-specific IgE (sIgE) testing and skin prick tests (SPT), are effective predictors of sensitivity to a specific allergen but not whether a patient will have allergy symptoms upon laboratory exposure [8]. In fact, the majority of children who test positive for either the blood-based sIgE tests or the SPT tests do not actually have FA [9].

The lack of effective IgE-mediated biomarkers for clinical FA [10] leads to the necessity of Oral Food Challenges (OFCs), the current gold standard for determining clinical reactivity [1][11]. However, these tests can be time-consuming, expensive, and potentially fatal, as they can induce anaphylaxis. As a result, food challenges arse often under performed, leading to an overdiagnosis of FA [9]. Sensitized patients are encouraged to develop immunity through consumption of the allergen, which cannot occur if they are incorrectly diagnosed as allergic. Thus, predictive models that can differentiate between food sensitization and clinical reactivity (*i.e.* true food allergy) are needed to avoid OFCs and reduce false positive diagnoses of FA.

Epigenetic factors have been noted to be a possible means of diagnosing food allergy [12][13]. Martino et al. 2015 [14] provides proof of principle that genome-wide levels of DNA Methylation, an epigenetic tag, are strong diagnostic markers of clinical FA. There is a high volume of recent FA research that uses DNAm: Martino et al. 2018 [15] used integrated DNAm and transcriptome profiling to conclude that the activation of naive CD4+ T cells results in poorer lymphoproliferative responses in children with FA, Sicherer and Sampson 2018 [16] suggest the use of DNAm signatures to create FA-related diagnostic tests, and Song et al. 2017 [17] state that DNAm regulates genes that are critical for the development of FA.

Martino et al. 2015 [14] pioneered the use of methylation data to create a diagnostic model for FA, achieving perfect classification when applying the shrunken centroid algorithm to a 96-CpG signature. To create their 96-CpG signature, Martino et al. 2015 [14] utilize *a priori* biological information, identifying overlapping protein-coding genes and enriching for immunologic genes.

This study analyzes the same dataset used by Martino et al. 2015 [14] with the following aims:

1. Replicating the perfect classification that was achieved in the previous work, while using substantially less than 96 features for the machine learning classifiers. In machine learning, simpler models (classifiers) are preferred over more complex ones, *ceteris paribus* [18][19].
2. Using a purely data-driven method to build a perfect classifier (*i.e.* one that does not use any *a priori* information), so that the methodology will be applicable to other diseases involving DNAm.
3. Extracting new biological insights by analyzing the set of genes associated with discriminating CpG features from the diagnostic classifiers.

All three of the aforementioned aims were met in this study.

## Materials and methods

Weka [20], a Java-based machine learning toolkit, was used for building the predictive models. The methods described here are also publicly-available at protocols.io (DOI: dx.doi.org/10.17504/protocols.io.x7pfrmn).

### Data

The research was conducted using a dataset found in the Gene Expression Omnibus (GEO) [21] under accession id GSE59999 [22]. The 71 patient samples in this dataset consisted of 29 patients with egg or peanut FA (tested positive on OFCs), 29 patients who were sensitized to one of those allergens but not food-allergic, and 13 patients who were neither sensitized nor allergic. Sensitized individuals tested positive for the skin prick tests but negative for the food challenge. The 58 allergic and sensitized samples were collected from infants who were between 11 to 15 months of age. Of the 29 sensitized patients, 16 were females and 13 were males; of the 29 allergic patients, 10 were females and 19 were males; and of the 13 non-allergic patients, 7 were females and 6 were males. Each of the non-allergic patients had reacted negatively to a skin prick test.

Similar to Martino et al. 2015 [14], the 13 patient samples who had neither FA nor sensitization were discarded, since the goal of the research was to build a classifier that distinguishes clinical FA from sensitization, and these 13 patients belonged to neither category. A skin prick test can be used in a clinical setting to filter out this non-allergic group.

Each sample consisted of normalized Methylation levels taken from mononuclear blood cells at 405,658 CpG islands across the genome. These Beta values were features for the machine-learning classifiers in this study.

### Splitting the dataset and creating independent folds

The 58 samples were randomly split into three cohorts: 40 samples for training, 10 samples for cross-validation, and 8 completely hidden samples for testing. Half of the samples in each of the three cohorts were allergic subjects, while the other half were sensitized. To avoid potential bias, eight random folds (K-Fold cross validation) [23] were created. In each fold, the samples were shuffled across the three cohorts such that each of the 58 samples was in the hidden dataset at least once across all 8 folds, as shown in S1 Table. All reported results are averaged over these eight independent folds, where the samples in the training, cross-validation, and hidden cohorts were varied. Each time, classifiers were re-trained on the new training set, the appropriate model was selected using the cross-validation set, and final accuracies were obtained on the hidden test set.

### Feature selection

DNAm datasets are characterized as having a small number of samples but a very high number of feature dimensions (HDLSS) [24]. To prevent overfitting and increase generalization, it is important to condense the feature list relative to the number of samples available. Computationally, it is very expensive to evaluate the more than 400K CpG features individually. Therefore, in order to limit the evaluation size and begin with a list of potentially highly-relevant CpG points, the NCBI GEO2R [25] tool was used to obtain a prioritized list of CpG features differentially methylated across the allergic and sensitized groups, using the forty training samples. This process was repeated for each of the eight folds. There are many freely-available R-based methods for generating this list of potentially highly-relevant features. The GEO2R tool uses the *limma* (Linear Models for Microarray Analysis [26]) R package for statistical analysis to identify differentially methylated features. Soneson and Delorenzi, 2013 provide a comparison of eleven freely-available R-based methods for differential expression analysis [27]. One or more of these methods could be used as an alternative to the GEO2R tool to generate this list of high-potential features.

The GEO2R tool produced eight ranked lists (one for each of the eight folds) of differentially methylated CpGs. The top 99 CpGs from each list were combined for an aggregate ranked list of 636 unique CpGs, the count being less than 792 since some of the CpGs overlapped across the eight folds. Table 1 shows fifteen of the top CpGs, where the ranking is a heuristic based on the position of the CpG in each list and its frequency of occurrence across lists. This ranking has no material significance in this methodology, since each of the unique 636 CpGs was later evaluated independently. It does, however, provide insight regarding comparisons between the features highlighted by GEO2R and those that appeared in the final CpG signature.

**Table 1:** Top CpGs and associated genes from GEO2R across 8 independent folds.

Rank	CpG	Associated Gene	Positions in Lists
1	cg06410630	*RNF213*;*LOC100294362*	6,1,1,21,91,3,13
2	cg13560030	*NTN4*	60,13,20,38,5,28
3	cg02681173	*LOC100190940*	2,35,1,16,56
4	cg09755579	*SNORA70B*;*USP34*	11,8,19,71,1
5	cg20502977	*COL6A3*	2,40,4,1
6	cg26124569	*LPP*	14,6,8,43
7	cg24616138	*CTBP2*	5,13,2,71
8	cg24584002	*RNASEH1*	20,18,40,32
9	cg03946731	*PKMYT1*	50,23,34,6
10	cg20463995	-	39,44,1,39,
11	cg09618933	-	48,12,60,5
12	cg10301401	*LMF1*	7,18,11
13	cg08378782	*RASGRP2*	9,27,38
14	cg21615831	*KSR1*	13,59,34,74
15	cg07060505	-	1,11,70

A CpG may not appear in the top 99 CpGs for all of the eight folds. The above ranking is based on the frequency of each CpG across the eight GEO2R lists as well as its ranking in each list. The order of the genes in this table has no methodological significance.

#### Hidden data accuracy score

For each fold, and for each of the 636 unique CpGs from the combined GEO2R list, four different machine learning classifiers were built: a Decision Tree (DT), Logistic Regression Model (LR), Radial Basis Function (RBF), and a Multi-Layer Perceptron (MLP). The perceptron was a deep learning network with an architecture of two hidden layers with ten nodes each. MLPs of other architectures can theoretically be added to the aforementioned four classifiers without changing the methodology, as model selection is not predetermined and instead stipulated by the cross-validation data.

Each predictive classifier used only one CpG and was built on the training data. For each fold and each CpG-feature, the classifier (DT, LR, RBF, or MLP) with the highest cross-validation accuracy was selected. Finally, the average hidden test set accuracy across the eight independent folds was computed. This quantity is referred to as the “accuracy score” throughout the rest of the paper. 636 of these accuracy scores (one for each CpG) and 20,352 classifiers (8 independent folds x 636 features x 4 classifiers) were created in total using this process for the single feature case.

### Increasing input features and classifier selection

Sequential forward feature selection (SFS) [28] was used to increase the number of input features until perfect classification was achieved. The top 18 CpGs found in the previous section (ranked by accuracy score) were combined two at a time, followed by three at a time, and so on until combinations of twelve were reached. Given the large number of potential combinations, each classifier was limited to a small subset of strong CpG-lists, to which a new input feature was added. On average, around 200 unique combinations were created for a given number of input features. Again, each unique input feature combination set was run 4 x 8 = 32 times to account for the four different classifier methods and 8 independent sample-distribution folds.

This process was stopped at twelve input-features, as perfect classification across the eight folds was achieved with 12-feature combinations. Adding additional features would increase the complexity of the classifiers, deviating from the objective of finding the simplest models to explain the data.

### Combining multiple classifiers using a simple voting scheme

Ensemble methods construct a set of machine learning classifiers and then classify new samples by taking a vote of the predictions from these models [29]. Ensemble systems generally perform better than their individual classifiers if each model has a better-than-random guessing chance and if the classifiers make diverse prediction errors [30].

While there are many ways to create an ensemble of classifiers, a simple majority-scheme method was used in this research. For simple-majority ensembles, odd numbers of classifiers are generally used to avoid the cases of ties [31] and to ensure a clear majority in the prediction from the independent classifiers. Therefore, odd numbers of classifiers, starting from 1 to 101, were combined using a simple voting scheme, i.e., each classifier independently predicted whether a sample was classified as FA or sensitized and the final prediction was the majority of predictions made across the different classifiers. The ensemble models were chosen based on their accuracy scores on hidden data (described above). An even number of classifiers could also have been used, with a default prediction of allergic in case of a tie in the predictions.

### Validating the CpG signature through dataset permutations

The final set of CpG signatures were re-validated through testing on a large number of datasets where the samples were repeatedly randomly allocated to the training, cross-validation, and hidden test sample cohorts. As earlier, the number of samples in train-validation-test datasets was kept at 40-10-8, with an equal number of allergy and sensitized samples in each cohort.

### Biological insights: Connecting to systems and pathways

Gene set analysis can provide biological context as well as insights into disease mechanisms and possible treatments [32][33]. Biological enrichment was performed by applying Illumina’s BaseSpace Correlation Engine [34] (BSCE) to the 13-gene list. To gain a deeper understanding of these genes, associated tissues and biological pathways were identified. The 13-gene list was also connected to Broad positional gene sets.

#### Connecting to Gene Ontology (GO)

Gene ontology concepts were used to identify functionally-related gene sets. [35] The Generic Gene Ontology (GO) Term Mapper [36] tool from Princeton University [37] was used to map granular GO annotations to a higher-level set of terms, thus providing a broad set of categories. REVIGO, an online tool that summarizes and visualizes lists of gene ontology terms, was also used to find a representative set of terms (through a clustering algorithm) [38][39].

## Results and discussion

### Single feature classifiers

**Fig 1:**
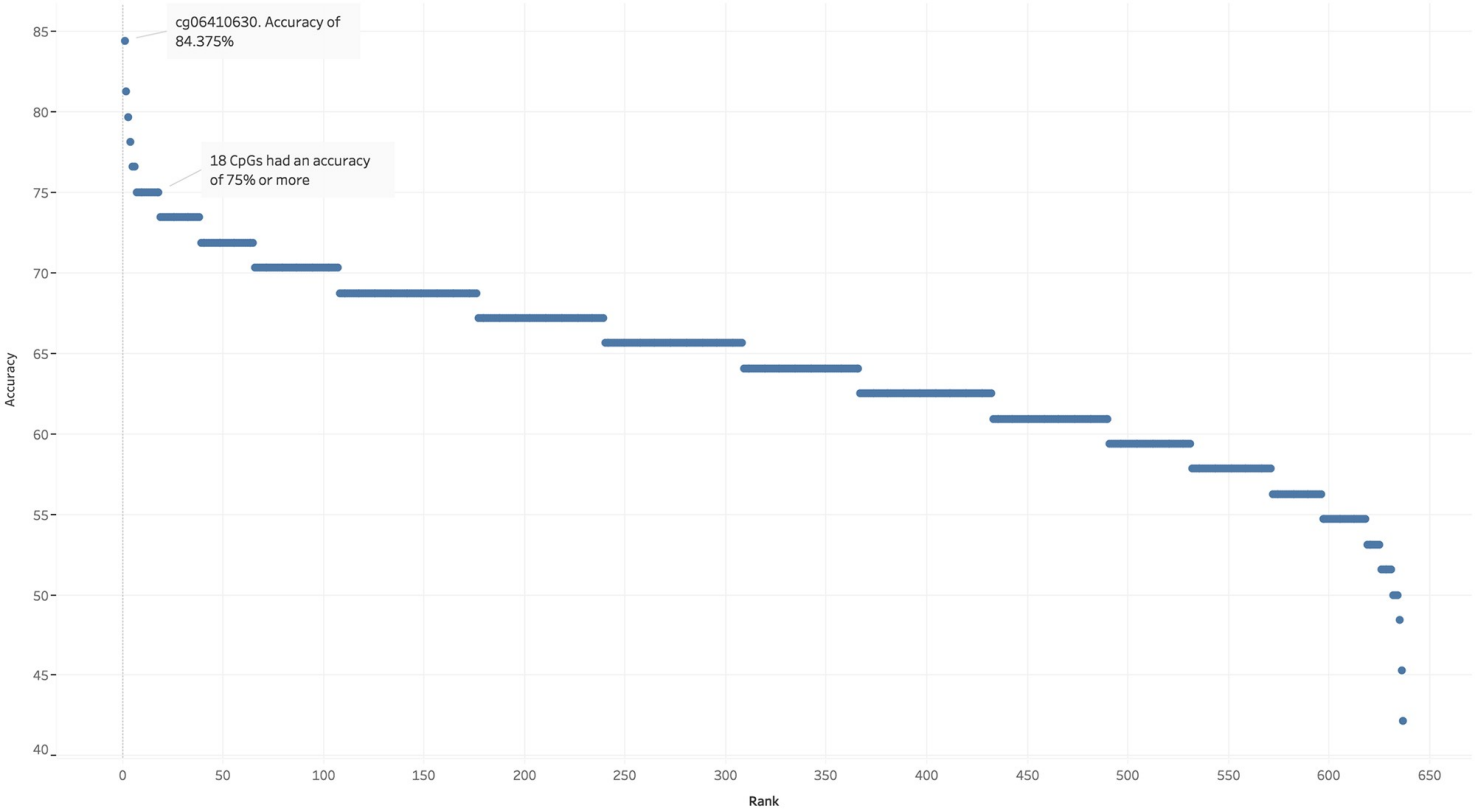
Average accuracy across eight independent folds for singular CpG features. The accuracy for each CpG is its average hidden-data accuracy across the 8 independent folds. cg06410630 was the strongest CpG biomarker with an average accuracy of 84.375%. 18 CpGs each had a score of 75% or more.

Fig 1 shows the distribution of the accuracy scores on hidden data for each of the 636 CpGs when they were the singular input feature for the machine learning classifiers. cg06410630, which maps to gene *RNF213*;*LOC100294362*, achieved an average accuracy of 84.375% and an average AUROC of 0.84, on the hidden test data across the eight independent folds. This CpG was also identified via the GEO2R analysis process. Table 2 shows the 18 CpGs that had an accuracy score of 75% or more, their associated genes, their accuracy scores, and their AUROC. Surprisingly, only two of the fifteen CpGs identified in Table 1 via GEO2R, namely cg06410630 and cg07060505, are in the list of eighteen shown in Table 2 that had the highest accuracy scores.

**Table 2:** Top CpGs and associated genes using a single input feature to a classifier across 8 independent folds.

Number	CpG	Gene	Average Accuracy	AUROC
1	cg06410630	*RNF213*;*LOC100294362*	84.375	0.8359375
2	cg06669701	*FAM190B*	81.25	0.7890625
3	cg06628000	*SARS*	79.6875	0.8359375
4	cg10461264	-	78.125	0.7421875
5	cg18988685	-	76.5625	0.8125
6	cg24616138	*CTBP2*	76.5625	0.7109375
7	cg27027230	*ARID5B*	75	0.765625
8	cg00936790	*KIF13B*	75	0.7421875
9	cg14414100	*SLC24A2*	75	0.7734375
10	cg00939931	*MAFK*	75	0.796875
11	cg06116095	*PANX1*	75	0.7421875
12	cg02788266	-	75	0.7734375
13	cg03068039	*ZNF252*;*TMED10P*	75	0.828125
14	cg25890092	*CD7*	75	0.8203125
15	cg19287711	-	75	0.78125
16	cg07033513	-	75	0.75
17	cg07060505	-	75	0.8125
18	cg26963090	*TIMP2*	75	0.7734375

These 18 CpGs achieved an accuracy score of 75% or higher when used as the singular feature in the machine learning classifiers. Their accuracy scores and AUROC were averaged over the 8 independent folds. For each fold, the machine learning classifiers were retrained and accuracy was computed on hidden test data.

### cg06410630 and cg06669701

**Fig 2:**
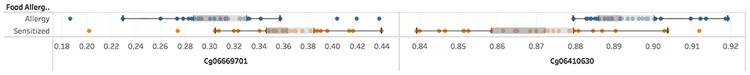
Distribution of methylation values for cg06410630 and cg06669701.

Fig 2 shows the methylation distributions of cg06410630 and cg06669701, the two best-performing CpGs, across the allergic and sensitized samples. Classifier models created using each one of these two CpGs as a singular input feature achieved an average hidden-data accuracy of 84.375% and 81.25%, respectively. As shown in Fig 2 the methylation values for cg06410630 are higher for food-allergic patients while the methylation values for cg06669701 are higher for food-sensitized patients.

### Combining classifiers via a voting scheme

**Fig 3:**
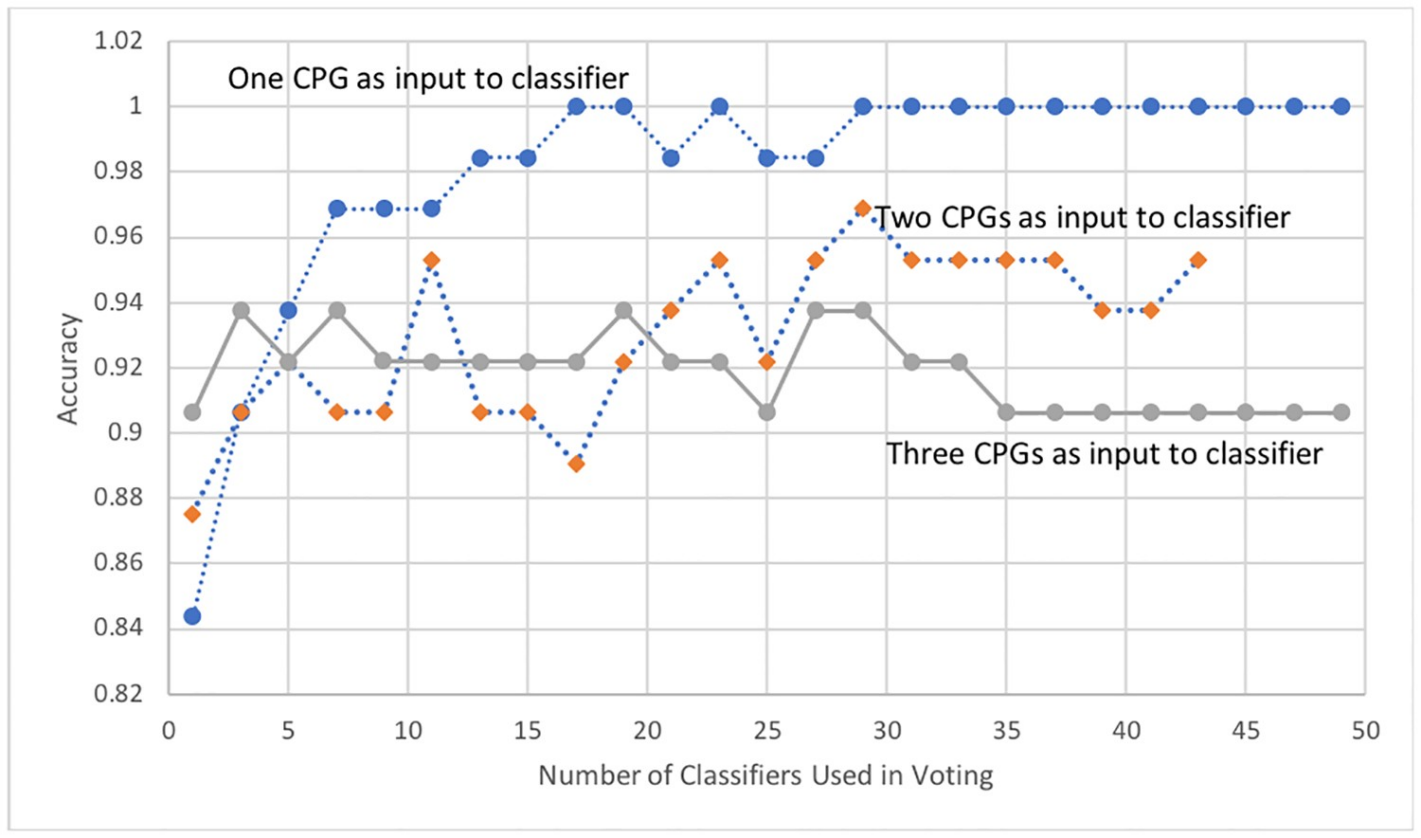
Average accuracy by combining multiple independent classifiers through a simple voting scheme. The graph shows the average accuracy achieved by combining classifiers with one to three CpG features through a majority voting scheme. Though the average accuracies for individual classifiers with single-feature CpG features are lower than those of classifiers with a larger number of CpGs, an ensemble (29 or more) of single-feature classifiers achieved perfect classification and outperformed ensembles of larger-feature classifiers.

When 29 or more independent single-feature classifiers were combined through the simple voting scheme, as shown in Fig 3, the accuracy scores on hidden data reached 100%. A possible explanation for this high-accuracy ensemble classification is provided in machine learning literature.

Dietterich 2000 [29] provides three main reasons on why ensemble models perform better than individual classifiers. Firstly, when the number of learning samples is small compared to the size of the hypothesis space—as is the case with this dataset—the learning algorithms can find many different hypotheses with the same accuracy from the training data. By constructing an ensemble out of these accurate classifiers, the ensemble can “average” out the votes and reduce the probability of selecting the wrong classifier. Secondly, machine-learning algorithms typically perform a local greedy search and may get stuck in a local minima. An ensemble created by classifiers that start their search from many different starting points may be more effective than a single classifier in finding the true function that describes the data. The third reason is related to how machine-learning problems are represented. When the sample size is small, a machine-learning algorithm develops only until the classifier can adequately represent the training dataset. Thus, the ensemble may collectively explore a wider set of possible hypotheses.

Fig 3 also shows the average accuracy achieved by combining independent classifiers with one, two, and three features. These multi-CpG classifiers were created from the list of the top 18 CpGs that obtained 75% accuracy or higher when used as singular input features (Section 2.4). It is speculated that the two, three, and four-CpG classifiers were not able to achieve as good of a combined ensemble classification (as compared to single-input ensemble classifiers) due to their non-independences, as some of the CpGs were repeated across the different classifiers in the ensemble—thus replicating potential prediction errors. However, 29 or more combined single-feature classifiers were diverse enough to yield perfect classification over the 8 hidden test sets.

### Deep learning classifiers dominate with complexity

**Fig 4:**
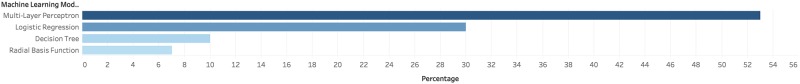
Distribution of machine learning classifier types for single-CpG feature models. The MLP was selected most frequently in the single-input case (53%), followed by Logistic Regression (30%), Decision Trees (10%), and Radial Basis Functions (7%). As the number of features per model increased, the MLP classifiers tended to further dominate the classifier selection process, with 86.67% of the twelve-feature classifiers attaining highest cross-validation accuracy with the MLP.

As shown in Fig 4, the deep learning network, an MLP with two hidden layers, was the most commonly selected classifier for the single CpG-feature case, followed by Logistic Regression, Decision Trees, and finally Radial Basis Functions. As the number of input features was increased, the MLP further dominated the classifier selection process: when combinations of 12-CpG features were used, around 86.67% of the classifiers had the highest cross-validation accuracy with the MLP (i.e. they “chose” the MLP classifier).

### Accuracy achieved using one to twelve CpGs as features

For each given number of features, from one to twelve, Table 3 shows the best (hidden) test accuracy achieved, the average accuracy of the top five classifiers (that had different feature sets), the average AUROC of the top five classifiers, and the best accuracy achieved through ensemble classification.

**Table 3:** Classifier statistics based on number of input features.

Features	Best Accuracy Score	Average Score Top 5	Average AUROC Top 5	Best Ensemble Accuracy	Steady-State EnsembleAccuracy
1	84.375	80	0.8031	**100**	**100**
2	87.5	86.25	0.9086	95.31	95.31
3	90.625	90.625	0.92815	92.1875	92.1875
4	93.75	93.75	0.9375	95.3125	95.3125
5	96.875	95.625	0.9468	96.875	96.875
6	95.3125	94.6875	0.9796875	96.875	96.875
7	96.875	96.25	0.9875	100	96.875
8	96.875	96.875	0.9890625	100	96.875
9	98.4375	97.5	0.99375	98.4375	98.4375
10	96.875	97.8125	0.996875	100	98.4375
11	98.4375	98.4375	0.9984375	98.4375	96.875
12	**100**	99.0625	**1**	**100**	**100**

The table shows the average 8-fold hidden accuracy (accuracy score) achieved by the best classifier for the given number of features. The third and fourth columns show the average accuracy score and AUROC for the top five classifiers, where each classifier has a different feature set. The fifth column shows the best score achieved by combining multiple independent classifiers via a simple voting scheme, and the sixth shows the steady-state (converging) accuracy score achieved by this combination after using 29+ independent classifiers.

#### Accuracy as a function of the number of input features

**Fig 5:**
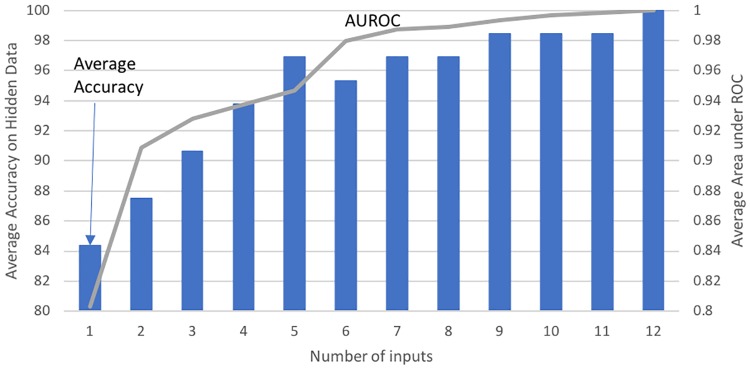
Best accuracy on hidden data and average AUROC as a function of the number of features. The bar graph shows the average accuracy on the hidden data achieved by the best individual classifier for a given number of CpG features, while the line graph shows the best average AUROC.

As shown in Fig 5 and in Table 3, the prediction accuracy of the classifier and the AUROC generally increase with the addition of a CpG feature. Perfect (100%) classification with an AUROC of 1 is obtained using twelve input CpGs. Perfect classification is not unusual for this dataset, as Martino et al. 2015 [14] also demonstrated the same accuracy using the shrunken centroid algorithm with 96 CpGs.

As discussed earlier and shown in Fig 3, combining 29 (or more) independent single-feature classifiers through a simple voting scheme resulted in perfect classification. Table 3 above shows the best accuracy achieved by combining independent classifiers for the given number of features and the steady-state accuracy achieved after many such classifiers (each with the same number of features) were combined. Perfect accuracy was achieved for two cases: twelve-feature classifiers and single-feature ensembles.

Table 4 contains the CpG features of the top two classifiers with twelve features. Note that eleven of the twelve CpGs for the two classifiers are the same, with cg00936790 and cg07033513 being the differing CpGs across the two classifiers. The deep learning classifiers created with each of these twelve-CpG feature sets achieved perfect hidden-data classification and also AUROCs of 1. Both results were averaged across the eight independent folds, as usual. S1 Table contains the details of the training, cross-validation, and hidden accuracies for this 12-feature case, as well as features for the top classifiers with 2-11 features.

**Table 4:** Top classifiers using twelve features averaged across 8 independent folds.

Number	CpG	Average Accuracy	AUROC
1	cg06410630, cg10461264, cg06116095, cg06628000, cg26963090, cg18988685, cg02788266, cg03068039, cg19287711, cg24616138, cg07060505, **cg00936790**	100%	1
2	cg06410630, cg10461264, cg06116095, cg06628000, cg26963090, cg18988685, cg02788266, cg03068039, cg19287711, cg24616138, cg07060505, **cg07033513**	100%	1

Eleven of the twelve CpGs were common for the two cases; cg00936790 and cg07033513 were the two CpGs that differed. Perfect classification, averaged on the eight completely hidden test cohorts, was achieved.

#### Heatmap of CpGs distribution across number of features

**Fig 6:**
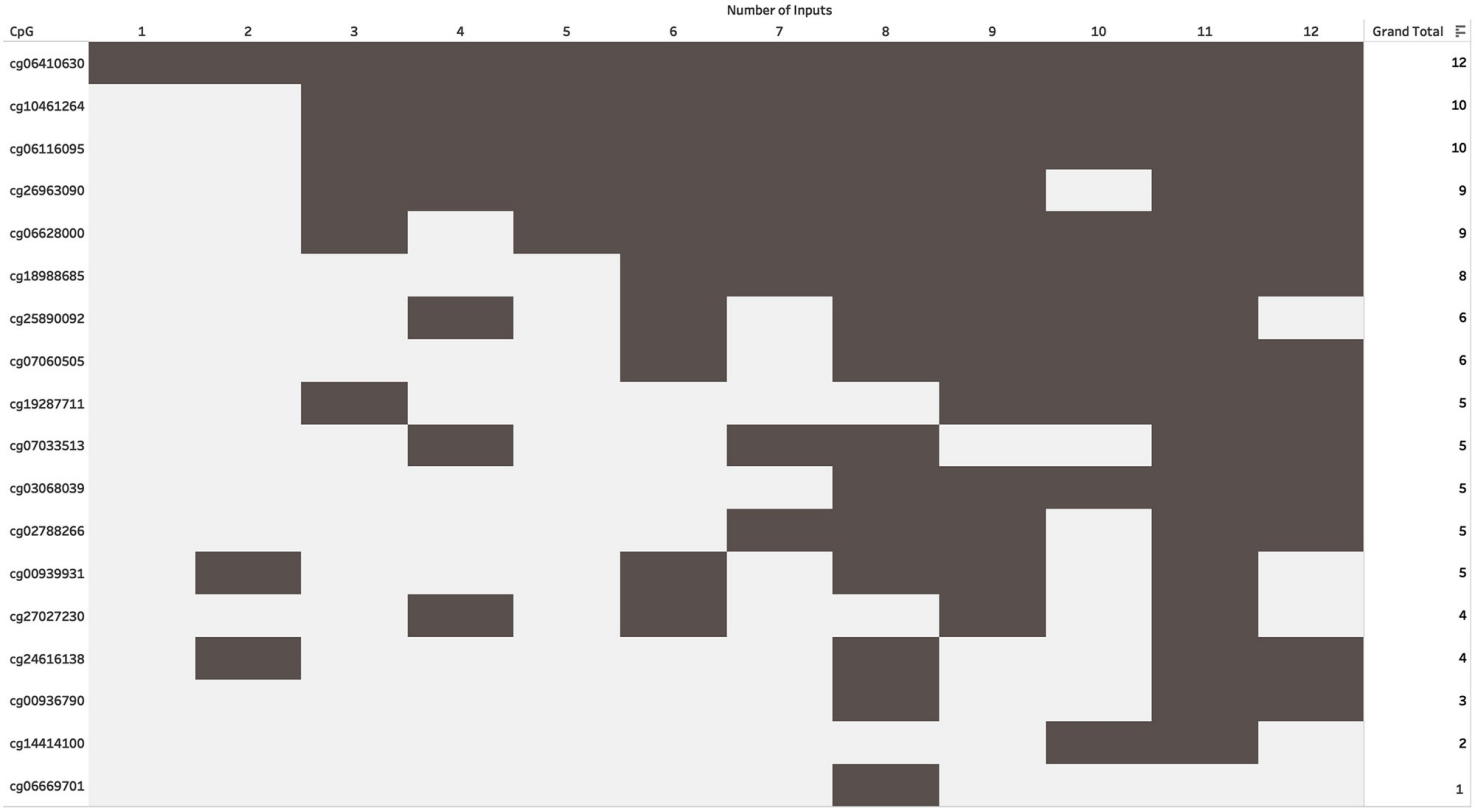
The plot shows the CpGs that appear in the classifiers with the highest accuracy for a given number of features. The shaded box indicates that the CpG appeared in the feature list of one of the best classifiers for that number of features. Note that at times there were multiple combinations of CpGs that achieved the same accuracy, due to which the number of shaded boxes may be more than the number of features.

Fig 6 shows the distribution of the different CpGs for the individual classifiers with the highest accuracy, for each number of input features. Note that many times multiple classifiers achieved the same highest accuracy (for a given number of features). As a result, the number of CpGs shown in the figure may be more than the specified number of features.

Interestingly, cg06410630 appeared in the best classifier for each of the feature-sizes from one to twelve. cg10461264, cg06116095, and cg26963090 were the next three most frequently appearing CpGs.

### Mapping CpGs to genes and creating a gene list

Taking the top 26 classifiers with 12 features each, including the two with the feature-lists enumerated in Table 4 as well as 24 additional 12-CpG classifiers that had an accuracy score of 98.4375% each, a list of 18 unique CpGs was created that mapped to 13 genes. The fact that CpGs across multiple genes were found to be strong biomarkers of FA indicates that FA is likely a polygenic disease. Table 5 contains the 18 CpGs found, their frequencies across the 26 different classifiers, their associated genes, and the biological descriptions of those genes. It also states whether each CpG was a novel association or if it was previously found by Martino et al. 2015 [14]. The top five CpGs appear in all 26 of the 12-feature classifiers. Interestingly, 7 of the 13 genes were also identified by Martino et al. 2015 [14], while six of the genes are unique to this study. The overlap of genes with those of Martino et al. 2015 [14], as well as the identification of additional genes, seems to validate the approach and findings of this study, since no *a priori* information was used in this completely data-driven approach.

**Table 5:** CpGs and associated genes from top 12-CpG classifiers.

	CpG	Frequency	Gene	Gene description	Identified Martino et al. [14]
1	cg06410630	26	*RNF213*; *LOC100294362*	Ring finger protein 213	Yes
2	cg06628000	26	*SARS*	Seryl-TRNA Synthetase	Yes
3	cg03068039	26	*ZNF252*; *TMED10P*	Zinc Finger Protein 252, Pseudogene Transmembrane P24 Trafficking Protein 10 Pseudogene 1	No
4	cg10461264	26	-		No
5	cg18988685	26	-		No
6	cg02788266	25	*ABCF2*	ATP Binding Cassette Subfamily F Member 2	No
7	cg26963090	22	*TIMP2*	TIMP Metallopeptidase Inhibitor 2	Yes
8	cg19287711	22	-		No
9	cg00939931	21	*MAFK*	MAF BZIP Transcription Factor K	Yes
10	cg25890092	17	*CD7*	CD7 Molecule	Yes
11	cg07060505	16	-		No
12	cg06116095	13	*PANX1*	Pannexin 1	Yes
13	cg24616138	13	*CTBP2*	C-Terminal Binding Protein 2	Yes
14	cg14414100	8	*SLC24A2*	Solute Carrier Family 24 Member 2	No
15	cg07033513	8	-		No
16	cg27027230	7	*ARID5B*	AT-Rich Interaction Domain 5B	No
17	cg00936790	7	*KIF13B*	Kinesin Family Member 13B	No
18	cg06669701	3	*FAM190B*	Coiled-Coil Serine Rich Protein 2	No

This table shows the frequency, associated genes, and gene descriptions of the 18 unique CpGs obtained from the 26 twelve-feature classifiers. The frequency shows the number of times each CpG was used across the 26 classifiers. Interestingly, seven of the thirteen genes identified in this study appeared in previous work conducted by Martino et al. 2015 [14]. The two pseudogenes [40], *ZNF252* and *TMED10P*, are counted as a single gene, resulting in a 13-gene signature.

#### Visualizing key CpGs

**Fig 7:**
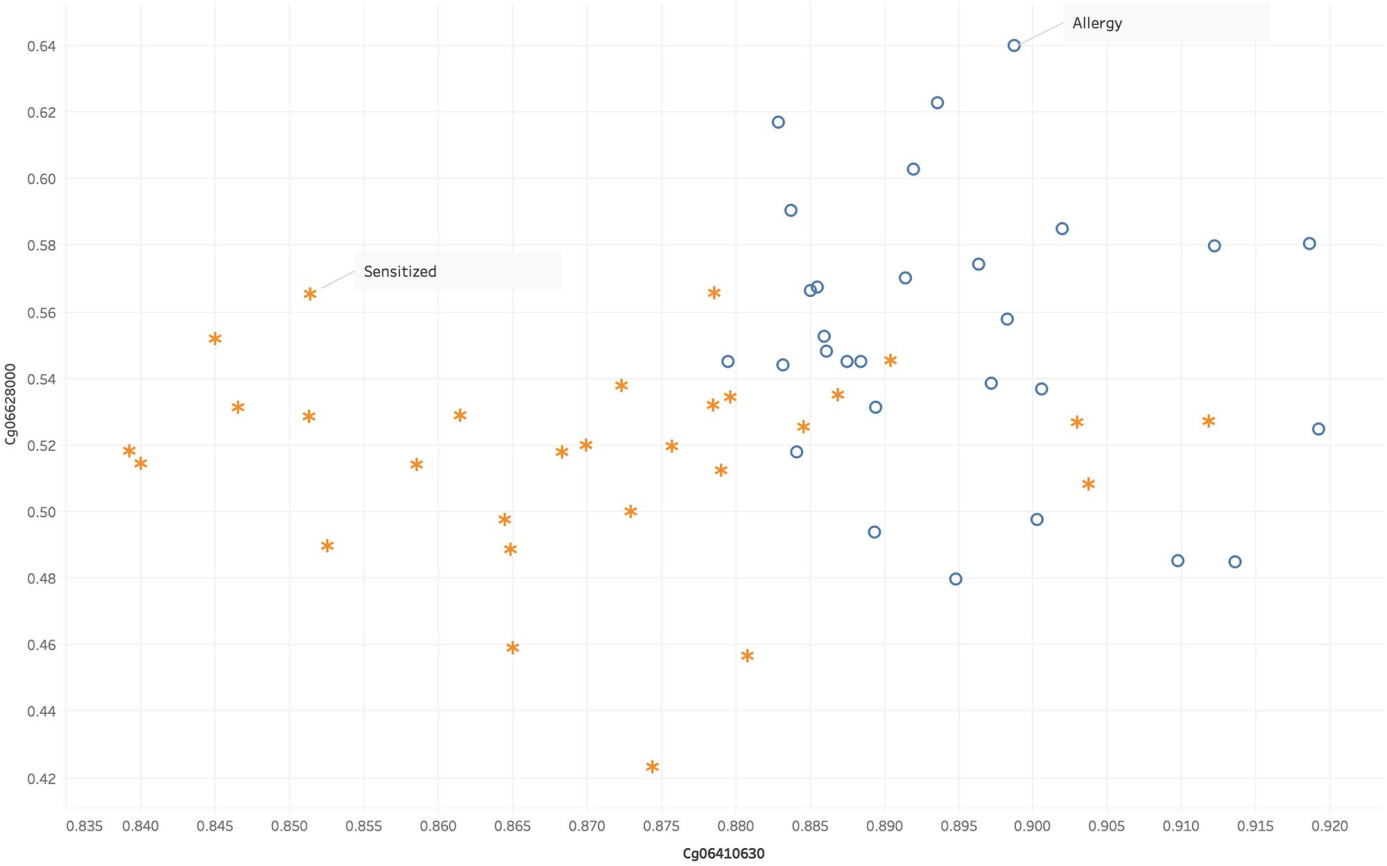
Plot of methylation values for cg06628000 versus cg06410630 for allergy and sensitized samples. The o markings denote allergy samples, while the * markings denote sensitized samples. There is some overlap in the middle region, while most other samples can be differentiated.

Fig 7 shows the methylation values of the top two CpGs, cg06628000 and cg06410630, plotted against each other for the FA and sensitized samples. There is some overlap between FA and sensitized values, but most of the samples can be separated using just these two features. As one can see from the plots, it is possible to differentiate between FA and sensitized samples using only small combinations of the features found in this study.

#### *RNF213* and *ABCF2*

cg06410630, which is associated with the gene *RNF213*, is the most discriminative CpG found in this study and a strong biomarker of FA. *RNF213* has previously been connected to immune response and virus defense [41]. The fact that *RNF213* has previously been associated with immune responses is significant, as FA is an immune-based disease. The CpG cg02788266 is sixth on the list and maps to the gene *ABCF2*. *ABCF2* is one of the ABC proteins, which transport various molecules across extracellular and intracellular membranes [42] and are associated with the immune system [43].

### Hidden data accuracy across many data permutations

To validate the diagnostic strength of the 18-CpG signature, the top 26 12-CpG classifiers were evaluated on a large number of hidden test sets, where the samples were repeatedly randomly allocated to the train-validation-test datasets. As shown in Table 6, the first two models achieved an average hidden-data accuracy of 95.3125% (AUROC 0.98328125) and 95.625% (AUROC 0.9853125). Similarly, the remaining 24 models averaged an accuracy of 94.15% to 95.625%. This high accuracy across a large number of randomly-generated dataset permutations further validates the strength of the 18-CpG signature.

**Table 6:** Average hidden data accuracy across a large number of dataset permutations.

Number	Signature	*n*	Average Accuracy	AUROC	95% CI for Accuracy
1	12-CpG #1	200	9.5.313	0.98328	(94.175, 96.451)
2	12-CpG #2	200	9.5.625	0.98531	(94.483, 96.767)
3	18-CpG	200	9.3.438	0.98047	(92.216, 94.734)

This table shows the average accuracy and AUROC across *n* randomized hidden test cohorts. The 95% Confidence Interval for accuracy is also shown and provides an estimate for the true population accuracy of each classifier on similar cohorts of patients.

### Biological insights: Connecting to biological systems and pathways

#### Gene expression in tissues

BSCE body atlas uses data from RNA-seq expression studies taken from the Genotype-Tissue Expression project (GTEx [44]).

The 13-gene signature was correlated to genes expressed in the Urogenital system (esp. Uterus), Respiratory system (esp. Lung), Digestive (Liver and Stomach fundus), Nervous System (Trigeminal ganglia and Dorsal root ganglia), Immune System (Thymus gland, Bone marrow, and Tonsil), and Endocrine System (Thyroid gland). The association of these genes with the respiratory, digestive, nervous, immune, and endocrine systems demonstrates the relevance of the thirteen genes with FA.

#### Canonical Wnt pathways

Canonical Wnt pathways “are involved in the control of gene expression, cell behavior, cell adhesion, and cell polarity” [45]. Twenty-seven canonical pathways were identified by BSCE for the 13-gene signature. The top four are listed below.

1. Oxidative Stress Induced Gene Expression Via Nrf2—*MAFK* is the common gene across the two gene sets. Nrf2 is associated with innate immunity [46].
2. Genes involved in The NLRP3 inflammasome—*PANX1* is the common gene across the two gene sets. NLRP3 inflammasome is associated with innate immunity [47].
3. Genes involved in Cytosolic tRNA aminoacylation—*SARS* is the common gene across the two gene sets. Cytosolic tRNA aminoacylation is also associated with the immune system [48].
4. Genes involved in Degradation of the extracellular matrix—*TIMP2* is the common gene across the two gene sets. This pathway is also associated with the immune system [49].

Interestingly, all four of these canonical pathways have been associated with the immune system. The gene *TIMP2* also mapped to the “ADAM 33 in asthma” canonical pathway [50].

#### Connecting to Gene Ontology (GO)

In GO, gene function is classified along three categories: molecular functions, cellular components, and biological processes. Table 7 shows the results from the GO enrichment analysis.

**Table 7:** Gene Ontology enrichment analysis.

	GO Annotation Data Set	Concept Number (Homo sapiens)
1	Biological process	3250
2	Molecular function	No statistically significant results
3	Cellular component	No statistically significant results

The 13-gene signature mapped to 3250 GO biological-process concepts, while there were no statistically significant matches for the molecular function and cellular component GO concepts. This match is based on GO Ontology database released on 2018-12-01 and was created through the GO Enrichment Analysis Tool [51].

S2 Table shows the top 37 GO terms that were mapped with at least one gene from the 13-gene signature using the Generic Gene Ontology Term Mapper [36]. The GO term “immune system process” (GO Id GO:0002376) is the seventh term in the 37-term list in S2 Table. This GO term is a direct child node of the “biological process” node and is defined as “Any process involved in the development or functioning of the immune system, an organismal system for calibrated responses to potential internal or invasive threats [52]” This is significant, as FA has been previously linked with the immune system.

Table 8 contains the results from the clustering using REVIGO [38][39], where the 37 terms have been clustered into 16 representative terms. The table highlights some of the representative terms that have been known to be associated with the immune system.

1. Two GO terms, signal transduction (GO:0007165) and response to stress (GO:0006950) cluster to a higher-order representation, “response to stress”. Stress has been associated with allergic and inflammatory disease, such as asthma, and its association with food allergy is a growing area of research [53].
2. The representative term “cytoskeleton organization” has three GO terms mapped to it extracellular matrix organization (GO:0030198), cellular component assembly (GO:0022607), and cytoskeleton organization (GO:0007010). The cytoskeleton plays an important role in innate immunity and cellular self-defense [54].
3. The “cell cycle” representative term has five GO terms mapped to it: cellular amino acid metabolic process (GO:0006520), cell-cell signaling (GO:0007267), cell cycle (GO:0007049), mitotic cell cycle (GO:0000278), and small molecule metabolic process (GO:0044281). The representative term “cell proliferation” has the term GO:0008283 mapped to it. The process of immune response is complex and dependent on the cell cycle. Immune response proceeds through different phases, from activation of lymphocytes, to rapid expansion by cell division, cell differentiation, stopping of cell division, and eventual death of most of the newly generated cells [55].
4. Homeostatic, associated with GO:0042592 and represented by the term “homeostatic process”, is the process of the body maintaining its internal environment, i.e., normal ranges for temperature, growth, and energy intake. The immune system, which fights foreign organisms such as bacteria, is the main system that maintains homeostasis [56].
5. Catabolism process, as represented by GO:0009056, breaks down complex substances into simpler ones with the production of energy. The immune system requires energy to counter pathogens, and this energy is obtained by catabolism of nutrients in activated immune cells [57].

**Table 8:** Gene Ontology terms summarization using clustering by Revigo [39].

	Representative Terms	GO Term (GO ID)	Uniqueness
1	anatomical structure development	aging (GO:0007568)	0.781
		anatomical structure development (GO:0048856)	0.781
2	biosynthesis	biosynthetic process (GO:0009058)	0.946
3	**catabolism**	catabolic process (GO:0009056)	0.936
4	**cell cycle**	cellular amino acid metabolic process (GO:0006520)	0.757
		cell-cell signaling (GO:0007267)	0.813
		cell cycle (GO:0007049)	0.813
		mitotic cell cycle (GO:0000278)	0.836
		small molecule metabolic process (GO:0044281)	0.858
5	**cell proliferation**	cell proliferation (GO:0008283)	0.894
6	**cytoskeleton organization**	extracellular matrix organization (GO:0030198)	0.762
		cellular component assembly (GO:0022607)	0.762
		cytoskeleton organization (GO:0007010)	0.777
7	growth	growth (GO:0040007)	0.944
8	**homeostatic process**	homeostatic process (GO:0042592)	0.924
9	**immune system process**	immune system process (GO:0002376)	0.944
10	locomotion	locomotion (GO:0040011)	0.944
11	neurological system process	neurological system process (GO:0050877)	0.944
12	protein targeting	cell motility (GO:0048870)	0.767
		transport (GO:0006810)	0.847
		transmembrane transport (GO:0055085)	0.848
		vesicle-mediated transport (GO:0016192)	0.865
		protein targeting (GO:0006605)	0.869
13	reproduction	reproduction (GO:0000003)	1
14	**response to stress**	signal transduction (GO:0007165)	0.778
		response to stress (GO:0006950)	0.911
15	symbiosis, encompassing mutualism through parasitism	symbiosis, encompassing mutualism through parasitism (GO:0044403)	0.944
16	tRNA metabolism	translation (GO:0006412)	0.827
		cellular protein modification process (GO:0006464)	0.853
		cellular nitrogen compound metabolic process (GO:0034641)	0.862
		tRNA metabolic process (GO:0006399)	0.868

The 37 GO terms were clustered into 16 representative terms using Revigo [38][39]. The concepts are sorted alphabetically using the representative terms. The GO terms within each representative term are sorted based on uniqueness, where smaller values denote higher uniqueness. The bolded representative terms have been known to be associated with the immune system.

Table 8 demonstrates a high association between the GO-based representative terms and the immune system.

#### Connecting to Broad positional gene sets

Broad positional gene sets correspond to each human chromosome and its chromosomal location or cytogenetic band that has at least one gene [58]. Three of the genes: *RNF213*, *TIMP2*, and *CD7*, are on chromosome 17 and intersect with chr17q25 [59]. The region chr17q25 has also been associated with psoriasis, a complex disorder of the skin and immune system [60]. Interestingly, Naumova et al. 2013 [61] identify sex- and age-dependent DNA methylation at the nearby 17q12-q21 locus to be associated with childhood asthma. Genes *ZNF252P* and *TMED10P1*, both located on chromosome 8, intersect with the chr8q24 [62]. The other positional gene sets overlapping with the 13-gene signature are as follows: chr1p13 (*SARS*), chr7p22 (*MAFK*), chr11q14 (*PANX1*), chr9p22 (*SLC24A2*), chr8p21 (*KIF13B*), chr10q26 (*CTBP2*), chr10q11 (*ARID5B*), and chr10q23 (*FAM190B*).

## Conclusion

### Accurate machine learning diagnostic classifiers

This research demonstrates a generalized data-driven machine learning approach to create accurate classifiers that distinguish between food-allergic and food-sensitized patients. By carefully adding a feature at a time (SFS) and leveraging two-layer-deep machine learning classifiers, two classifiers of twelve features each were created that achieved perfect classification on hidden data, averaged across eight independent folds in which the training, testing, and cross-validation samples were varied.

Interestingly, perfect classification was also achieved when 29 or more single-feature classifiers (an ensemble of classifiers) were combined using a voting scheme. This single-feature ensemble requires significantly less computational effort to derive than the process of building higher-dimension classifiers and may prove useful for other DNAm datasets. Additionally, simpler machine learning classifiers, such as those used in this study with 12-input features, are preferred *ceteris paribus*.

The final 18-CpG list was re-validated on a large number of dataset permutations, where the samples in the training, cross-validation, and test groups were shuffled. The 18-CpG signature and the 26 12-CpG signatures (subsets of the 18) consistently achieved around 94% to 96% accuracy. This high accuracy, similar to that achieved by previous work on this dataset, is better than any known clinical test today [14][63][64][65].

### 13-Gene signature and biological enrichment

The 18-CpG list mapped to a novel 13-gene signature that is a strong biomarker of FA. Out of these 13 genes, seven genes overlapped with the genes found by Martino et al. 2015 [14], while the remaining six were unique. The identified genes are expressed in the Urogenital, Endocrine, Digestive, Immune, and Nervous Systems. The genes also mapped to a number of canonical Wnt pathways, GO, and positional gene sets. These genes and pathways merit further research for potential therapeutic applications. Many of the genes were also identified with various aspects of the immune system, validating these findings since FA is an immune-based disease. Moreover, the fact that such a few number of CpGs (12) achieved high accuracy implies the strong associations of those genetic loci with FA.

### Generalizable data-driven approach: Application to other diseases

The methods used in this study, being completely data-driven, are applicable to other problems that use High Dimension Low Sample Size (HDLSS) data. This methodology can be used with DNAm data to gain new biological insights and create highly-accurate classifiers for diseases such as certain cancers, Asthma, Crohn’s disease, and HIV [66]. The applicability of this methodology to other diseases is even more significant due to the invention of the microarray [67] and the Illumina Infinium BeadChip, which have made high-throughput processing of DNAm data easier [68] and more accessible.

Because they do not use *a priori* information, the classifiers used in this study can computationally evolve as new data are added, increasing in accuracy as time progresses. Additionally, the ensemble approach using single-feature classifiers could provide computationally efficient diagnostic classifiers for DNAm data.

### Limitations and future work

Since not all of the possible CpG feature combinations have been exhaustively evaluated, it is possible that there is a CpG signature with a smaller number of features that can perfectly classify the examples for this dataset. However, that approach is computationally expensive and may not yield significant additional biological insights.

A greater limitation for both this research and future food allergy-related work is the lack of publicly-available datasets and the low number of samples associated with FA. Classifiers generally improve with data, especially when the number of features is large [69]. Having more DNAm FA-related data would validate and further increase the generalizability of the diagnostic classifiers created in this study. Validation of the 13-gene signature in a second cohort would also be of tremendous value. Additionally, since methylation values can change with age [70], it will be insightful to evaluate the 18-CpG signature on an older cohort, as this dataset consisted of 11-15 month infants.

Furthermore, as the data used in this study contained both peanut-allergic and egg-allergic patients, future work should analyze the differences between the DNAm underpinnings for the two allergens. Arasi et al. 2018 [71] also call out the need for researchers to build algorithms for diagnosing FA by integrating data from different sources and technologies, and Tham and Leung 2018 [72] point out that the mechanisms of FA may differ in different global populations. Thus, evaluating different DNAm datasets associated with FA may provide additional unique insights.

Future work should be focused on creating clinical tests for distinguishing between FA and sensitized patients, thus helping avoid misdiagnosis and dangerous OFCs. The genes and pathways highlighted by this research should also be further studied to elucidate the mechanisms and possible treatments of food allergies. This data-driven machine-learning approach opens the door to the computational analysis of other diseases, which may lead to enhanced research and understanding of those ailments.

## Supporting information

S1 Table Various classifier details. (PDF)

S2 Table GO terms from the biological process ontology. (PDF)

S1 Fig Distribution of methylation values for cg06628000, cg03068039, and cg18988685. (PDF)
