# Correction: Machine learning approach yields epigenetic biomarkers of food allergy: A novel 13-gene signature to diagnose clinical reactivity

**DOI:** 10.1371/journal.pone.0220470

**Published:** 2019-07-24

**Authors:** The PLOS ONE Staff

In the Introduction, there is an error in the third sentence of the third paragraph. The correct sentence is: As a result, food challenges are often under performed, leading to an overdiagnosis of FA [9].

There is an error in Table 6. The vales in column 4 "Average Accuracy" are incorrect. The publisher apologizes for the error. Please see the correct Table 6 here.

**Table 6:** Average hidden data accuracy across a large number of dataset permutations.

Number	Signature	*n*	Average Accuracy	AUROC	95% CI for Accuracy
1	12-CpG #1	200	95.313	0.98328	(94.175, 96.451)
2	12-CpG #2	200	95.625	0.98531	(94.483, 96.767)
3	18-CpG	200	93.438	0.98047	(92.216, 94.734)

This table shows the average accuracy and AUROC across *n* randomized hidden test cohorts. The 95% Confidence Interval for accuracy is also shown and provides an estimate for the true population accuracy of each classifier on similar cohorts of patients.
