# Supplementary material for: Machine learning approach yields epigenetic biomarkers of food allergy: A novel 13-gene signature to diagnose clinical reactivity
Source: PLoS One. 2019 Jun 19;14(6):e0218253. doi: 10.1371/journal.pone.0218253 (PMC6584060; doi:10.1371/journal.pone.0218253)
Supplement: S1 Table — (PDF) [file pone.0218253.s001.pdf]

**Table 1. Sample distribution across the eight independent folds**

| Fold | Cross-Validation Samples (10) | Hidden Test Samples (8) |
|---|---|---|
| 1 | GSM1463332,GSM1463337,GSM1463380,GSM1463387, GSM1463389,GSM1463340,GSM1463351,GSM1463357, GSM1463370,GSM1463383 | GSM1463334,GSM1463346,GSM1463347, GSM1463348,GSM1463350,GSM1463356, GSM1463358,GSM1463382 |
| 2 | GSM1463328,GSM1463343,GSM1463363,GSM1463378, GSM1463398,GSM1463339,GSM1463367,GSM1463371, GSM1463382,GSM1463386 | GSM1463331,GSM1463337,GSM1463338, GSM1463341,GSM1463349,GSM1463352, GSM1463379,GSM1463384 |
| 3 | GSM1463334,GSM1463337,GSM1463372,GSM1463387, GSM1463394,GSM1463336,GSM1463345,GSM1463346, GSM1463368,GSM1463386 | GSM1463342,GSM1463343,GSM1463344, GSM1463363,GSM1463371,GSM1463383, GSM1463388,GSM1463396 |
| 4 | GSM1463332,GSM1463363,GSM1463376,GSM1463378, GSM1463380,GSM1463339,GSM1463358,GSM1463368, GSM1463382,GSM1463397 | GSM1463354,GSM1463357,GSM1463361, GSM1463386,GSM1463387,GSM1463390, GSM1463392,GSM1463393 |
| 5 | GSM1463330,GSM1463361,GSM1463384,GSM1463389, GSM1463398,GSM1463331,GSM1463338,GSM1463348, GSM1463351,GSM1463367 | GSM1463328,GSM1463346,GSM1463349, GSM1463353,GSM1463365,GSM1463368, GSM1463372,GSM1463380 |
| 6 | GSM1463328,GSM1463354,GSM1463363,GSM1463372, GSM1463389,GSM1463340,GSM1463341,GSM1463353, GSM1463377,GSM1463397 | GSM1463330,GSM1463332,GSM1463344, GSM1463351,GSM1463370,GSM1463371, GSM1463378,GSM1463385 |
| 7 | GSM1463328,GSM1463350,GSM1463356,GSM1463372, GSM1463380,GSM1463331,GSM1463338,GSM1463340, GSM1463351,GSM1463390 | GSM1463339,GSM1463345,GSM1463352, GSM1463362,GSM1463389,GSM1463391, GSM1463397,GSM1463398 |
| 8 | GSM1463337,GSM1463363,GSM1463380,GSM1463389, GSM1463398,GSM1463331,GSM1463349,GSM1463357, GSM1463388,GSM1463393 | GSM1463336,GSM1463340,GSM1463367, GSM1463373,GSM1463376,GSM1463377, GSM1463384,GSM1463394 |

k-fold cross validation using k=8. Each sample appears at least once in the hidden set. In addition, for every fold there are 40 training samples that are not shown in the table. These 40 training samples are the remaining samples out of the total 58 that are in neither the cross-validation nor the hidden test set for that fold. For each fold, there are an equal number of food-allergic and food-sensitized samples in each of the training, cross-validation, and hidden sample sets.

**Table 2. Accuracy details for the twelve feature cases averaged across 8 independent folds**

| Classifier Id | Training Accuracy | Cross-Validation Accuracy | Hidden Test Accuracy |
|---|---|---|---|
| 1 | 100% | 96.25% | 100% |
| 2 | 100% | 95% | 100% |

The two 12-feature classifiers achieved 100% accuracy on the training and test datasets and 96.25% and 95% on the cross-validation dataset. Further details are in Table 3 below.

**Table 3. Accuracy details for the twelve feature cases for each of the eight independent folds**

| Classifier Id | Fold | Classifier Type | Training Accuracy | CV Accuracy | CV Sensitivity | CV Specificity | Test Accuracy |
|---|---|---|---|---|---|---|---|
| 1 | 1 | MLP | 100% | 100% | 100% | 100% | 100% |
| | 2 | MLP | 100% | 100% | 100% | 100% | 100% |
| | 3 | MLP | 100% | 100% | 100% | 100% | 100% |
| | 4 | MLP | 100% | 80% | 71% | 100% | 100% |
| | 5 | RBF | 100% | 90% | 83% | 100% | 100% |
| | 6 | MLP | 100% | 100% | 100% | 100% | 100% |
| | 7 | MLP | 100% | 100% | 100% | 100% | 100% |
| | 8 | MLP | 100% | 100% | 100% | 100% | 100% |
| 2 | 1 | MLP | 100% | 100% | 100% | 100% | 100% |
| | 2 | MLP | 100% | 90% | 83% | 100% | 100% |
| | 3 | MLP | 100% | 100% | 100% | 100% | 100% |
| | 4 | MLP | 100% | 90% | 83% | 100% | 100% |
| | 5 | RBF | 100% | 90% | 83% | 100% | 100% |
| | 6 | MLP | 100% | 90% | 83% | 100% | 100% |
| | 7 | MLP | 100% | 100% | 100% | 100% | 100% |
| | 8 | LR | 100% | 100% | 100% | 100% | 100% |

Details of the accuracy achieved by the two 12-feature classifiers across the eight folds for the training, cross-validation (CV), and hidden samples. MLP is a multi-layer perceptron with two hidden layers of 10 nodes each. RBF stands for radial basis function and LR stands for Logistic Regression. All cross-validation errors were false negatives. The sensitivity and specificity for the training and hidden dataset is 100%.

**Table 4. Top 2-CpG classifiers**

| Classifier Id | CpG Features | Average Hidden Accuracy | Average AUROC |
|---|---|---|---|
| 1 | cg06410630, cg24616138 | 87.5 | 0.91796875 |
| 2 | cg06410630, cg00939931 | 87.5 | 0.9296875 |
| 3 | cg06628000, cg19287711 | 85.9375 | 0.9296875 |
| 4 | cg18988685, cg27027230 | 85.9375 | 0.8359375 |

cg06410630 appeared in both of the models that achieved an average accuracy of 87.5% on the eight hidden data folds.

**Table 5. Top 3-CpG classifiers**

| Classifier Id | CpG Features | Avg. Hidden Accuracy | Avg. AUROC |
|---|---|---|---|
| 1 | cg06410630, cg10461264, cg26963090 | 90.625 | 0.9453125 |
| 2 | cg06410630, cg10461264, cg06116095 | 90.625 | 0.9375 |
| 3 | cg06410630, cg10461264, cg19287711 | 90.625 | 0.9140625 |
| 4 | cg06410630, cg10461264, cg06628000 | 90.625 | 0.90625 |

cg06410630 and cg10461264 appeared in all four of the 3-feature classifiers that achieved an average accuracy of 90.625% on the eight hidden data folds.

**Table 6. Top 4-CpG classifiers**

| Classifier Id | CpG Features | Avg. Hidden Accuracy | Avg. AUROC |
|---|---|---|---|
| 1 | cg06410630, cg10461264, cg25890092, cg06116095 | 93.75 | 0.9375 |
| 2 | cg06410630, cg10461264, cg26963090, cg27027230 | 93.75 | 0.9453 |
| 3 | cg06410630, cg10461264, cg06116095, cg07033513 | 93.75 | 0.9219 |
| 4 | cg06410630, cg10461264, cg27027230, cg06116095 | 93.75 | 0.9609 |

cg06410630 and cg10461264 appeared in all four of the top 4-feature classifiers, while cg06116095 appeared in three of the four and cg27027230 appeared in two of the four.

**Table 7. Top 5-CpG classifiers**

| Classifier Id | CpG Features | Avg. Hidden Accuracy | Avg. AUROC |
|---|---|---|---|
| 1 | cg06410630, cg10461264, cg06116095, cg06628000,cg26963090 | 96.875 | 0.9453 |
| 2 | cg06410630, cg06116095, cg10461264, cg06628000, cg18988685 | 95.3125 | 0.9609 |
| 3 | cg06410630, cg10461264, cg25890092, cg06628000, cg26963090 | 95.3125 | 0.9453 |

cg06410630, cg10461264, and cg06628000 appeared in all three of the 5-feature classifiers while cg06116095 and cg26963090 appeared in two of the three classifiers.

**Table 8. Top 6-CpG classifiers**

| Classifier Id | CpG Features | Avg. Hidden Accuracy | Avg. AUROC |
|---|---|---|---|
| 1 | cg06410630, cg10461264, cg06116095, cg06628000, cg26963090, cg25890092 | 95.3125 | 0.984375 |
| 2 | cg18988685, cg00939931, cg07060505, cg10461264, cg06410630, cg06628000 | 95.3125 | 1 |

cg06410630, cg10461264, and cg06628000 appeared in both of the top 6-feature classifiers with an average accuracy of 95.3125% across the eight hidden data folds.

**Table 9. Top 7-CpG classifiers**

| Classifier Id | CpG Features | Avg. Hidden Accuracy | Avg. AUROC |
|---|---|---|---|
| 1 | cg06410630, cg10461264, cg06116095, cg06628000, cg26963090, cg18988685, cg02788266 | 96.875 | 0.9921875 |
| 2 | cg18988685, cg00939931, cg07060505, cg10461264, cg06410630, cg06628000, cg03068039 | 96.875 | 0.9921875 |
| 3 | cg18988685, cg00939931, cg07060505, cg10461264, cg06410630, cg06628000, cg07033513 | 96.875 | 1 |

cg06410630, cg10461264, cg06628000, and cg18988685 appeared in all three of the top 7-feature classifiers, which had an accuracy of 96.875%.

**Table 10. Top 8-CpG classifiers**

| Classifier Id | CpG Features | Avg. Hidden Accuracy | Avg. AUROC |
|---|---|---|---|
| 1 | cg06410630, cg10461264, cg06116095, cg06628000, cg26963090, cg06669701, cg18988685, cg03068039 | 96.875 | 0.96875 |
| 2 | cg18988685, cg00939931, cg07060505, cg10461264, cg06410630, cg06628000, cg03068039, cg25890092 | 96.875 | 1 |
| 3 | cg06410630, cg10461264, cg06116095, cg06628000, cg26963090, cg18988685, cg02788266, cg24616138 | 96.875 | 0.9921875 |
| 4 | cg18988685, cg00939931, cg07060505, cg10461264, cg06410630, cg06628000, cg03068039, cg00936790 | 96.875 | 0.9921875 |
| 5 | cg06410630, cg10461264, cg06116095, cg06628000, cg26963090, cg18988685, cg02788266, cg25890092 | 96.875 | 0.9921875 |
| 6 | cg18988685, cg00939931, cg07060505, cg10461264, cg06410630, cg06628000, cg06669701, cg03068039 | 96.875 | 0.984375 |
| 7 | cg18988685, cg00939931, cg07060505, cg10461264, cg06410630, cg06628000, cg03068039, cg07033513 | 96.875 | 0.9921875 |

cg06410630, cg10461264, cg06628000, and cg18988685 appeared in all seven of the top 8-feature classifiers.

**Table 11. Top 9-CpG classifiers**

| Classifier Id | CpG Features | Avg. Hidden Accuracy | Avg. AUROC |
|---|---|---|---|
| 1 | cg06410630, cg10461264, cg06116095, cg06628000, cg26963090, cg18988685, cg02788266, cg03068039, cg19287711 | 98.4375 | 1 |
| 2 | cg18988685, cg00939931, cg07060505, cg10461264, cg06410630, cg06628000, cg03068039, cg25890092, cg27027230 | 98.4375 | 1 |

cg06410630, cg10461264, cg06628000, cg18988685, and cg03068039 appear in both the 9-feature classifiers.

**Table 12. Top 10-CpG classifiers**

| Classifier Id | CpG Features | Avg. Hidden Accuracy | Avg. AUROC |
|---|---|---|---|
| 1 | cg18988685, cg00939931, cg07060505, cg10461264, cg06410630, cg06628000, cg03068039, cg25890092, cg19287711, cg14414100 | 98.4375 | 1 |
| 2 | cg18988685, cg00939931, cg07060505, cg10461264, cg06410630, cg06628000, cg03068039, cg25890092, cg27027230, cg06116095 | 98.4375 | 0.9921875 |

cg18988685, cg00939931, cg07060505, cg10461264, cg06410630, cg06628000, cg03068039, and cg25890092 were common across both the 10-feature classifiers.

**Table 13. Top 11-CpG classifiers**

| Classifier Id | CpG Features | Avg. Hidden Accuracy | Avg. AUROC |
|---|---|---|---|
| 1 | cg06410630, cg18988685, cg06628000, cg03068039, cg10461264, cg00939931, cg07060505, cg25890092, cg19287711, cg14414100, cg26963090 | 98.4375 | 1 |
| 2 | cg06410630, cg18988685, cg06628000, cg03068039, cg10461264, cg00939931, cg07060505, cg25890092, cg19287711, cg14414100, cg02788266 | 98.4375 | 1 |
| 3 | cg06410630, cg18988685, cg06628000, cg03068039, cg10461264, cg26963090, cg00939931, cg07060505, cg25890092, cg19287711, cg02788266 | 98.4375 | 0.9921875 |
| 4 | cg06410630, cg18988685, cg06628000, cg03068039, cg10461264, cg00939931, cg07060505, cg25890092, cg19287711, cg02788266, cg07033513 | 98.4375 | 0.9921875 |
| 5 | cg06410630, cg18988685, cg06628000, cg03068039, cg10461264, cg00939931, cg07060505, cg25890092, cg19287711, cg14414100, cg07033513 | 98.4375 | 1 |
| 6 | cg06410630, cg18988685, cg06628000, cg03068039, cg10461264, cg26963090, cg00939931, cg07060505, cg19287711, cg02788266, cg06116095 | 98.4375 | 1 |
| 7 | cg06410630, cg18988685, cg06628000, cg03068039, cg10461264, cg00939931, cg07060505, cg25890092, cg19287711, cg02788266, cg24616138 | 98.4375 | 0.9921875 |
| 8 | cg06410630, cg18988685, cg06628000, cg03068039, cg10461264, cg00939931, cg07060505, cg25890092, cg06116095, cg27027230, cg00936790 | 98.4375 | 0.9921875 |
| 9 | cg06410630, cg18988685, cg06628000, cg03068039, cg10461264, cg26963090, cg19287711, cg02788266, cg24616138, cg06116095, cg00936790 | 98.4375 | 0.9765625 |
| 10 | cg06410630, cg18988685, cg06628000, cg03068039, cg10461264, cg26963090, cg00939931, cg19287711, cg02788266, cg24616138, cg06116095 | 98.4375 | 0.9921875 |
| 11 | cg06410630, cg18988685, cg06628000, cg03068039, cg10461264, cg26963090, cg19287711, cg02788266, cg24616138, cg07033513, cg06116095 | 98.4375 | 0.9921875 |
| 12 | cg06410630, cg18988685, cg06628000, cg03068039, cg10461264, cg26963090, cg00939931, cg25890092, cg02788266, cg06116095, cg27027230 | 98.4375 | 1 |

12 models were found that achieved an average hidden data accuracy of 98.4375% using 11 features. cg06410630, cg18988685, cg06628000, cg03068039, and cg10461264 were common across all 12 models.
