# Supplementary material for: Machine learning approach yields epigenetic biomarkers of food allergy: A novel 13-gene signature to diagnose clinical reactivity
Source: PLoS One. 2019 Jun 19;14(6):e0218253. doi: 10.1371/journal.pone.0218253 (PMC6584060; doi:10.1371/journal.pone.0218253)
Supplement: S2 Table — (PDF) [file pone.0218253.s002.pdf]

Table 1. GO terms from the biological process ontology

	GO Term	GO ID	Genes Annotated to the GO Term	GO Term Usage in Gene List	Genome Frequency (out of 19497 annotated genes)
1	signal transduction	GO:0007165	*ARID5B*, *CD7*, *CTBP2*, *KIF13B*, *RNF213*, *TIMP2*	6 of 12 genes, 50.00%	6030, 30.93%
2	anatomical structure development	GO:0048856	*ARID5B*, *CD7*, *CTBP2*, *KIF13B*, *RNF213*, *TIMP2*	6 of 12 genes, 50.00%	5588, 28.66%
3	biosynthetic process	GO:0009058	*ARID5B*, *CTBP2*, *MAFK*, *SARS*	4 of 12 genes, 33.33%	6416, 32.91%
4	cell differentiation	GO:0030154	*ARID5B*, *CTBP2*, *KIF13B*, *TIMP2*	4 of 12 genes, 33.33%	3981, 20.42%
5	cellular nitrogen compound metabolic process	GO:0034641	*ARID5B*, *CTBP2*, *MAFK*, *SARS*	4 of 12 genes, 33.33%	6622, 33.96%
6	transport	GO:0006810	*KIF13B*, *PANX1*, *SLC24A2*, *TIMP2*	4 of 12 genes, 33.33%	4956, 25.42%
7	**immune system process**	**GO:0002376**	*CD7*, *KIF13B*, *TIMP2*	3 of 12 genes, 25.00%	2974, 15.25%
8	cell-cell signaling	GO:0007267	*PANX1*, *RNF213*, *SLC24A2*	3 of 12 genes, 25.00%	1593, 8.17%
9	cellular protein modification process	GO:0006464	*ARID5B*, *RNF213*, *TIMP2*	3 of 12 genes, 25.00%	4113, 21.10%
10	cellular component assembly	GO:0022607	*PANX1*, *RNF213*	2 of 12 genes, 16.67%	2713, 13.91%
11	protein-containing complex assembly	GO:0065003	*PANX1*, *RNF213*	2 of 12 genes, 16.67%	1686, 8.65%
12	anatomical structure formation involved in morphogenesis	GO:0048646	*RNF213*, *SARS*	2 of 12 genes, 16.67%	1020, 5.23%
13	response to stress	GO:0006950	*MAFK*, *PANX1*	2 of 12 genes, 16.67%	3830, 19.64%
14	cell proliferation	GO:0008283	*CTBP2*, *TIMP2*	2 of 12 genes, 16.67%	1973, 10.12%
15	transmembrane transport	GO:0055085	*PANX1*, *SLC24A2*	2 of 12 genes, 16.67%	1534, 7.87%
16	catabolic process	GO:0009056	*RNF213*, *TIMP2*	2 of 12 genes, 16.67%	2443, 12.53%
17	reproduction	GO:0000003	*ARID5B*	1 of 12 genes, 8.33%	1391, 7.13%
18	cytoskeleton-dependent intracellular transport	GO:0030705	*KIF13B*	1 of 12 genes, 8.33%	157, 0.81%
19	nervous system process	GO:0050877	*SLC24A2*	1 of 12 genes, 8.33%	1364, 7.00%
20	protein targeting	GO:0006605	*KIF13B*	1 of 12 genes, 8.33%	397, 2.04%
21	homeostatic process	GO:0042592	*SLC24A2*	1 of 12 genes, 8.33%	1641, 8.42%
22	cellular amino acid metabolic process	GO:0006520	*SARS*	1 of 12 genes, 8.33%	367, 1.88%
23	extracellular matrix organization	GO:0030198	*TIMP2*	1 of 12 genes, 8.33%	335, 1.72%
24	cytoskeleton organization	GO:0007010	*FAM190B*	1 of 12 genes, 8.33%	1212, 6.22%
25	translation	GO:0006412	*SARS*	1 of 12 genes, 8.33%	604, 3.10%
26	mitotic cell cycle	GO:0000278	*TIMP2*	1 of 12 genes, 8.33%	976, 5.01%
27	small molecule metabolic process	GO:0044281	*SARS*	1 of 12 genes, 8.33%	2081, 10.67%
28	vesicle-mediated transport	GO:0016192	*TIMP2*	1 of 12 genes, 8.33%	2000, 10.26%
29	symbiont process	GO:0044403	*CTBP2*	1 of 12 genes, 8.33%	841, 4.31%
30	cell motility	GO:0048870	*ARID5B*	1 of 12 genes, 8.33%	1488, 7.63%
31	cell morphogenesis	GO:0000902	*KIF13B*	1 of 12 genes, 8.33%	932, 4.78%
32	tRNA metabolic process	GO:0006399	*SARS*	1 of 12 genes, 8.33%	188, 0.96%
33	chromosome organization	GO:0051276	*ARID5B*	1 of 12 genes, 8.33%	1174, 6.02%
34	aging	GO:0007568	*TIMP2*	1 of 12 genes, 8.33%	284, 1.46%
35	locomotion	GO:0040011	*ARID5B*	1 of 12 genes, 8.33%	1721, 8.83%
36	growth	GO:0040007	*ARID5B*	1 of 12 genes, 8.33%	922, 4.73%
37	cell cycle	GO:0007049	*TIMP2*	1 of 12 genes, 8.33%	1793, 9.20%

The Generic Gene Ontology (GO) Term Mapper tool was used to to map granular GO annotations to a broader, higher-level set of terms. Note that two genes, *ZNF252* and *TMED10P*, did not map to any GO terms. The "immune system process" term is of particular interest since FA has been previously linked with the immune system.
