# Supplementary material for: Machine learning approach yields epigenetic biomarkers of food allergy: A novel 13-gene signature to diagnose clinical reactivity
Source: PLoS One. 2019 Jun 19;14(6):e0218253. doi: 10.1371/journal.pone.0218253 (PMC6584060; doi:10.1371/journal.pone.0218253)
Supplement: S1 Fig — (PDF) [file pone.0218253.s003.pdf]

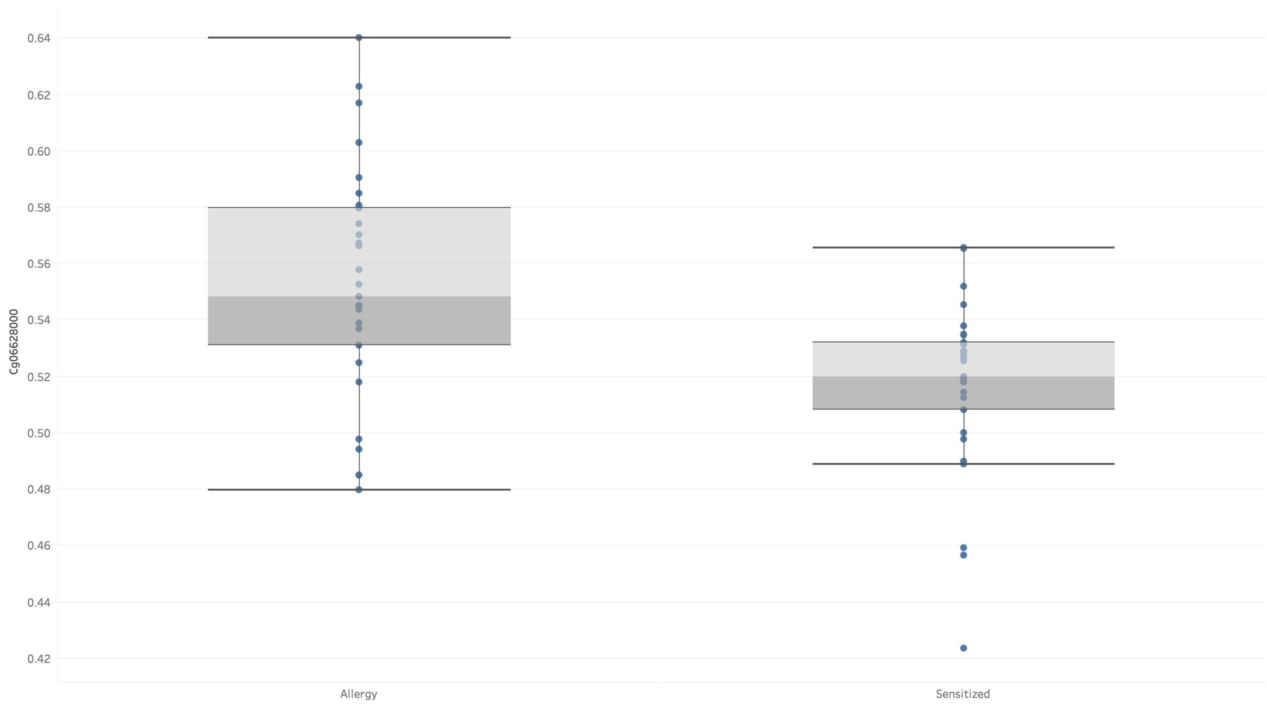

Figure 1. Distribution of methylation values for cg06628000. The methylation values are on average higher for samples associated with FA.

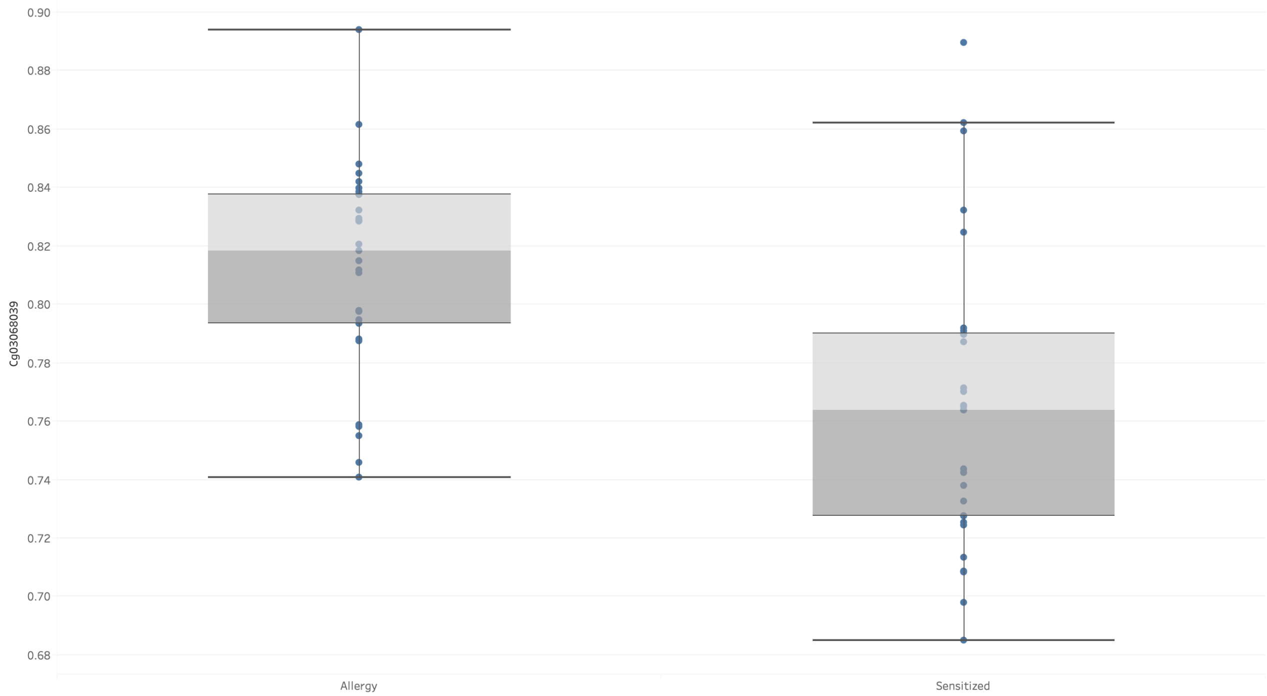

Figure 2. Distribution of methylation values for cg03068039. The methylation values are on average higher for samples associated with FA.

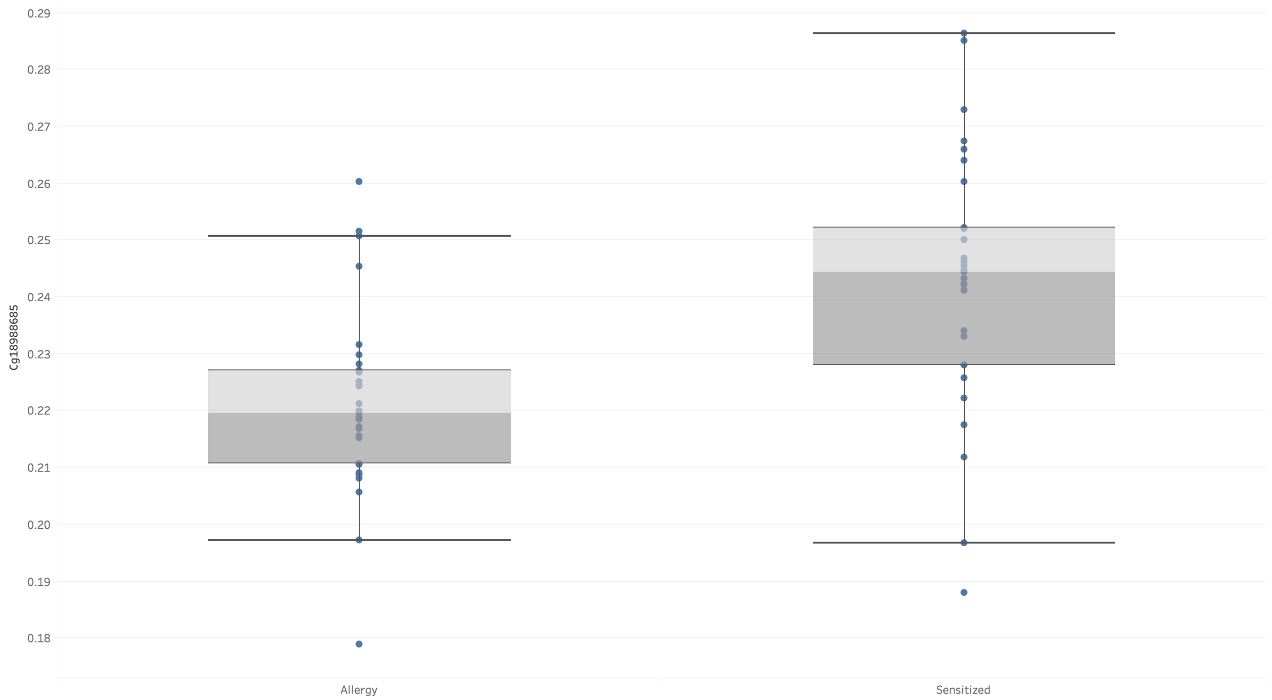

Figure 3. Distribution of methylation values for cg18988685. The methylation values are on average higher for samples associated with sensitized samples.
